# Emotional Experience of *Caam^2^* in Teaching: Power and Interpretation of Teachers’ Work

**DOI:** 10.3389/fpsyg.2016.01400

**Published:** 2016-09-13

**Authors:** Kwok K. Tsang, Tsun L. Kwong

**Affiliations:** ^1^Faculty of Education, Chinese University of Hong KongHong Kong, China; ^2^Division of Business and Management, BNU-HKBU United International CollegeZhuhai, China

**Keywords:** teachers’ work, appraisal, interpretation, power, emotions

## Abstract

The study explores the social psychological process of teachers’ emotional experiences. Twenty-one secondary schoolteachers in Hong Kong were interviewed. The findings show that the teachers generally felt *caam^2^* (a Cantonese adjective that covers a range of meanings like gloomy, dreadful, tragic, pitiful, pathetic, and miserable) in teaching. The social psychological process of the emotional experience of *caam^2^* involves how teachers interpret the significance of their actual work in attaining the teaching goal of making a difference. If they interpret their work as incapable of fulfilling the goal, they will experience negative emotions in teaching. The findings also suggest that the interpretation is affected by teachers’ power which is unequally distributed according to teachers’ teaching experience and managerial roles.

## Introduction

“It’s like I have to work around the clock every day. Even though I don’t go to school at weekends, I still need to mark students’ assignments at home. Of course, I don’t prefer such practice, but the workload is simply overflowing. Well… it’s like I have lost my private time. Since I am always occupied with my work, I can never say ‘yes’ when my friends asked me out for the weekend.” (Jack^1^)

The above excerpt from Jack, a secondary schoolteacher in Hong Kong, is a typical comment shared by many teachers in Hong Kong about their working condition. From the excerpt, we can see how workload has been exhausting to the teachers. Studies have reported that the actual working hours for Hong Kong teachers, on average, are in the range of 10–12 per day and 50–60 per week (e.g., Lee et al., 2007; Morris and Adamson, 2010). Literatures about well-being of Hong Kong teachers have critiqued that such a working condition may lead to teachers’ negative emotional experiences, such as anxiety, depression, and burnout, which have negative impacts on teachers’ psychological well-being (Mo, 1991; Pearson and Moomaw, 2005; Lee et al., 2007; Yeung and Liu, 2007; Lau et al., 2008; Chan et al., 2010; Fung, 2012; Chan, 2013; Hassard et al., 2016). However, The Committee on Teachers’ Work (2006) finds that heavy workload is not an issue if Hong Kong teachers perceived themselves to be contributing to students’ learning. In other words, there may be a social psychological process of teachers’ emotional experiences. As we shall see, the social psychological process may be related to teachers’ power and interpretations of teachers’ work, but there is little attention paid to it in the literature. Thus, the study seeks to fill the research gap and provide recommendations to improve teachers’ emotional experiences.

In a past decade, the Hong Kong government has devoted attention and effort to improve the quality of secondary school education by reforming the school management and curriculum (Chan, 2010). Researchers observe that these continuous education reforms have created high pressure and exhaustion among the secondary schoolteachers resulting in negative emotional experience of the teachers (Cheng, 2009; Chen, 2016; Tsang and Kwong, 2016). Thus, a study of the secondary schoolteachers may provide opportunities to understand the social psychological process of teachers’ emotional experiences.

## Appraisal Theory of Emotions

In recent years, appraisal theory provides a theoretical framework to study teachers’ emotional experiences (Frenzel, 2014). The theory suggests that appraisals are individuals’ cognitive judgments about situations and events, and these judgments are the primary cause of emotions (Schutz et al., 2009). Accordingly, teachers’ emotional experiences are the results of teachers’ subjective appraisals of the situations and events in teaching (Frenzel, 2014). Within the framework of appraisal theory, the central appraisals for teachers’ emotions are goal congruence and conduciveness, coping potential, accountability, and goal significance (Beck et al., 2015). From the list of central appraisals, Frenzel et al. (2009) note that goals play an important role in the arousal of teachers’ emotions. They suggest that teachers tend to experience positive emotions if teachers consider a situation as congruent with and/or conductive to the attainment of their significant goal (goal congruence and conduciveness), if they have resources to reach a significant goal (coping potential), and/or if they have no responsibility for the non-attainment of their significant goal (accountability); otherwise, teachers may experience negative emotions.

Frenzel et al. (2009) suggest that the significant goals of teachers are the instructional goals which are broadly categorized as the follows. First, cognitive goal refers to students’ acquisition of subject-specific competencies. Second, motivational goal concerns with students’ motivational engagement in classroom activities and learning content. Third, social-emotional goal relates to students’ development of social and emotional competencies such as respect for others and empathy. Finally, relational goal deals with the establishment of good teacher-student relationship.

Since the nature of the instructional goals is related to students, studies have shown that students are the antecedent of teachers’ emotions (Lortie, 1975; Hargreaves, 1999; Frenzel, 2014). If the teachers interpret the students’ classroom behaviors as matching or contributing to the lesson goals, or if the teachers interpret that they are obliged to the attainment of certain important lesson goals and these goals are ultimately attained by their efforts, the teachers will experience positive emotions; otherwise, they will experience negative emotions (Frenzel, 2014).

Accordingly, the social psychological process of teachers’ emotional experiences is the appraisal process by which teachers interpret whether or not, or how possible their significant goals in teaching can be attained. However, there are some limitations in the appraisal theory. First, the theory only refers the significant goals to the instructional goals. However, instructional goals are only one type of goals in teaching and may not necessarily be the most significant to teachers. Many teachers indicate that they choose to become teachers because of intrinsic values, such as the desire to make a difference, or extrinsic values, such as job security and financial rewards (Ballantine and Hammack, 2013). Studies illustrate that when teachers achieve these values, they will experience intensive positive emotions, such as job, pride, and excitement, and a sense of self-fulfillment (Lortie, 1975; Hargreaves, 1999; Bullough, 2011). In other words, these values may be more salient and significant goals than the instructional goals to teachers in teaching. Therefore, we should explore which goals the teachers are usually committed to and how the goals influence the teachers’ emotional experiences.

Second, the appraisal theory does not recognize the social dimension of teachers’ emotional experiences. Human emotions are socially constructed (Turner, 2007). That means how we feel is influenced by the social situations which pre-define how we should feel (Hochschild, 1979). In other words, how teachers feel should not be only affected by their interpretation of goal attainment or non-attainment, but also determined by their social situations. Thus, Schutz et al. (2009) suggest that research on teachers’ emotions should investigate how teachers’ emotions are constructed by the transaction between teachers and their social situations. Otherwise, our understandings of teachers’ emotions will be confined (Saunders, 2013).

## Social Constructionism of Emotions

In order to overcome the limitations of the appraisal theory, the present study adopts the perspective of social constructionism on emotions. Under this perspective, emotions are self-reflexive experiences directly linked to a situation in which they occur (Lupton, 1998). This means that emotions are aroused by the meanings social actors give to the self or the situation through interpretation of the self or the situation (Denzin, 1984). In this sense, understanding teachers’ emotional experiences needs an investigation of how teachers interpret themselves or the social situation in which they work. To achieve this, Lortie (1975) recommends researchers to focus on teachers’ perspective on their goals or purposes in teaching because it reflects the meaning teachers give to themselves and their situation in teaching. Research has shown that prospective and current teachers generally commit themselves to the goal of making a difference (Lam, 2011; Ballantine and Hammack, 2013), because the role of teachers is socially defined as being responsible, interested, and enthusiastic to nurture students’ growth (Hansen, 1998). In order to attain the goal, the teachers try their best to do everything to benefit students’ learning and development (Maclure, 1993). If they interpret that they have successfully attained the goal, they may see themselves as good and competent teachers and feel positive; otherwise, they may regard themselves as bad and incompetent teachers and feel negative (Kelchtermans, 2011).

Nevertheless, different from the appraisal theory, social constructionism does not simply concur with the explanation that teachers’ emotional experiences are just the result of teachers’ interpretation of whether their significant goals in teaching are attained. It further suggests that the interpretations and the resulted emotional experiences are conditioned by social situations (Turner, 2007). For example, when a work situation forces teachers to do much work resulting in a lack of time for preparation of lessons or catering of students’ learning needs, the teachers may perceive much of their work as non-instructional, even though much of these perceived non-instructional work (e.g., organizing extracurricular activities to students) carries instructional values (Tsang and Kwong, 2016). As a result, the teachers feel frustrated, demoralized and even alienated from the work, because they tend to consider most of their work as deleterious to attaining one of the significant goals to make a difference (Santoro, 2011). Why do the teachers not see the instructional values of their work in the situation above? One possible reason is about power.

## Power

In addition to the interpretation and the nature of the significant goals, the social psychological process of teachers’ emotional experience may also be related to power. Literature has shown that power is a variable affecting human’s emotions (Turner, 2011). In the literature, the concept of power is generally synonymous with the concept of control. That means having power can control issues, decisions, behaviors or individuals in a relationship (Ingersoll, 2003). In organizational settings, power is structurally and unequally distributed according to the organizational hierarchy. Those who occupy higher administrative positions (administrators) will have stronger power than those whose administrative positions are lower (employees). The unequal distribution of power will then separate employees’ conception from execution (Braverman, 1974). It means that the employees are forced to do much work without understanding the values of their work, because they are powerless to both decide and design their work (Braverman, 1974). As a result, the employees are prone to negative emotional experiences such as meaninglessness and self-estrangement, because they may perceive their work as purposeless (Blauner, 1964).

Apple (1986) argues that the power issue is the same among teaching profession. Many teachers are the front-line classroom teachers who are subordinated to school administrators in the organizational hierarchy of the school. Teachers are generally forced to do much work by their school administrators (Ball, 2003). Since most of the time the teachers think that the imposed work is contradictory to their teaching goals, such as making a difference (Santoro, 2011), they therefore tend to feel negative toward their work.

It is noted that some teachers are appointed to take up more important managerial roles than other teachers in schools. In other words, these teacher–administrators have stronger power than the other teachers in school, so they may experience less negative emotions than other teachers. For example, Rinehart and Short (1994) find that teachers who have managerial roles tend to be more satisfied with their work than those who do not. According to Wong (1997), the teacher–administrators are more able to understand the values of the work imposed by the school since they can influence teachers’ work and working condition by participating in the decision-making process of the school. Therefore, they tend to be more satisfied when struggling with the different work tasks.

Thus, the emotional experience of teachers who have managerial roles tends to be more positive than that of teachers who do not have managerial roles, because the former type of teachers have stronger power that enables them to better understand the value of the work assigned by the school.

## Materials and Methods

The above discussion implies that the social psychological process of teachers’ emotional experience may involve teachers’ interpretations and power. Accordingly, the process may involve both the meanings teachers give to their work and the social situation that affects the meaning-making. Therefore, this study adopted an in-depth interview method for data collection. This is because the in-depth interview method empowers the researchers to explore and discover the meanings and process which constitute the phenomenon being studied through a more thorough investigation of the participants’ perspectives, feelings, interpretations, and social interactions with reference to social contexts (Seidman, 2006).

### Participants

There were three stages of sampling. First, one of the researchers invited secondary schoolteachers to participate in the study via his social network. In this stage, six teachers with less than 6 years of teaching experience were individually interviewed. These teachers were temporary teachers (1-year contract) without managerial roles. After a brief analysis of the interview data, the researchers wondered if the findings were applicable to the more experienced and tenure teachers with managerial roles. Therefore, in the secondary stage, the researchers asked the six participants to refer their colleagues who had more teaching experience and held managerial roles in the schools for the study. As a result, an additional seven teachers were individually interviewed. After a brief analysis of the interview, the researchers identified that most of the participants taught language and art subjects, such as English, Chinese, History, and Liberal Studies. It was wondered whether the findings were applicable to those teachers who taught science and other subjects. Moreover, the researchers noted that most of the participants taught in Band three schools. The secondary schools are divided into three types – Band one, Band two, and Band three – in Hong Kong according to the merit of the students. Band one schools consist of the best one-third of the total number of students in Hong Kong in terms of academic performance, while Band three schools consist of the worst one-third. Band two schools consist of the remaining one-third of students whose academic performance is average. In other words, the findings up to this stage might only reflect the teachers’ situations in the underperforming (Band three) schools rather than the best (Band one) and average (Band two) schools. Thus, in the third stage of sampling, the researchers asked the participants to refer their friends who taught other subjects or worked in the Band one and Band two schools to the study. The sampling ended when data saturation occurred. Eventually, a total of 21 Hong Kong secondary schoolteachers were interviewed (**Table 1**).

**Table 1:** Participants’ profile.

	Age	Contract type	Managerial role	Subject category	School type (school)
**Early-career (≤6 years)**					
Amy	31	Temporary	None	Language Arts	Band two (A)
Crystal	28	Temporary	None	Language	Band two (C)
				Arts	
Olivia	29	Temporary	None	Language	Band two (D)
Mandy	26	Tenure	None	Business/Economics	Band three (E)
				Arts	
Emma	31	Temporary	None	Language Arts	Band two (A)
Ken	27	Tenure	None	Sciences	Band one (I)
Bonny	30	Temporary	None	Sciences	Band three (E)
				Business/Economics	
				Arts	
Leo	26	Temporary	None	Sciences	Band three (E)
Peter	27	Temporary	None	Arts	Band three (H)
**Mid-career (9–20 years)**					
Jack	36	Temporary	None	Language	Band three (B)
Isabella	34	Tenure	Subject panel head	Arts	Band three (E)
Eva	37	Tenure	Subject panel head	Sciences	Band three (F)
Tom	34	Tenure	Subject panel head	Language	Band three (G)
				Arts	
John	39	Tenure	Subject panel head	Business/Economics	Band three (G)
				Arts	
Flora	35	Tenure	None	Language	Band three (G)
Rex	42	Tenure		Arts	Band one (J)
**Late-career (25–40 years)**					
David	59	Tenure	Committee/team leader	Language	Band three (G)
Paul	46	Tenure	Subject panel head	Business/Economics	Band three (G)
				Arts	
Connie	51	Tenure	Committee/team leader	Language	Band three (G)
Sally	50	Tenure	Subject panel head	Language	Band three (G)
Sam	49	Tenure	Committee/team leader	Sciences	Band three (F)

### Procedure

All interviews were semi-structured and tape-recorded. During the interviews, the participants were asked to introduce themselves briefly on the subjects they were teaching, their age, teaching position, and teaching experience. After that, they were asked the questions listed in **Table 2**.

**Table 2:** Interview questions.

(1) What kinds of duties are you responsible for in your school?
(2) Would you mind describing your working conditions?
(3) How do you feel about your work and your working conditions?
(4) Would you mind telling me the reasons why you teach?
(5) Would you mind telling me about some of your emotional experiences at work during your teaching career?

During the interviews, the question sequence was flexibly organized. Generally, each interview began with questions one and two, because for these questions, the participants were just required to give straightforward descriptions with minimal recall, judgment and interpretation (Seidman, 2006). In many cases, in response to question two, the participants would express their feelings about their work or teaching conditions, especially the negative ones. Question three aimed to follow-up on how the teachers felt about, and interpreted, their work. Question five aimed to obtain a more balance and comprehensive picture of teachers’ emotional experience. Other questions were then asked for clarification and to elicit more information about the participants’ experience. If the participants did not share thoughts about their interpretation of teachers’ work, they would be asked question four in order to explore their general reasons for starting the teaching career. Each participant had been interviewed for 1.5 hours on average.

The interviews took place between February and June 2012. It should be noted that this was a busy period in the typical academic year for secondary schoolteachers in Hong Kong. During this period, teachers had to prepare the final year high school students for a public examination scheduled in April and May. Moreover, many Hong Kong secondary schools arranged their school final examinations in June, so teachers were under a lot of stress as they were required to prepare school examination papers in advance during the period of the interview exercise in the current study. Furthermore, the secondary schools might also carry out teacher appraisal in February and March, so teachers might have to spend much time and energy preparing for the appraisal. Thus, the participants in the present study might have been very busy, exhausted, and under stress during the data collection period. All this might have caused them to express more negative emotions toward their work during the interviews.

### Data Analysis

After data collection, all the interviews were transcribed. The data was coded by open coding and then focus coding with NVivo7. We developed a tentative coding scheme to analyze the data at the very beginning in order to facilitate the data analysis process (Bogdan and Biklen, 2007). The tentative coding scheme contained the following codes: interpretation, emotions, and power. Throughout the analysis process, we kept refining and modifying the coding scheme in order to improve the credibility of data analysis by comparing incidents in data with other incidents, incidents with themes, and themes with other themes during the coding process (Glaser and Strauss, 1967). Finally, we identified the themes of *caam^2^*, workload, and interpretations of teachers’ work emerged from the data. From the data, we also identified that there were three sub-themes of interpretations of teachers’ work, including making a difference, instructional work, and non-instructional work. In addition to these attempts, we employed the technique of member checking which is a means to improve the credibility of data analysis (Bogdan and Biklen, 2007).

In addition, to explore whether there were differences in teachers’ emotional experience and interpretations of teachers’ work between teachers, a comparison of the similarities and differences between the narratives of the participants differing in teaching experiences and managerial roles was done by running matrix coding with NVivo7.

### Researcher Reflexivity

In this study, some of the participants were friends or ex-colleagues of one of the authors, and the other participants were the friends referred by the author’s friends or ex-colleagues. Therefore, there was a possibility of teacher–participants with similar emotional experiences in teaching and perceptions toward their work and work situation. Therefore, the findings may be biased to some extent. Nevertheless, the sampling strategy might have an advantage for the study in obtaining a more realistic and truthful data (Esterberg, 2002; Corbin and Strauss, 2008; Creswell, 2009). Since most of the participants were the authors’ friends or referred friends, it was easy for the authors to develop close and trustful relationships with the participants. Such a relationship might make the participants feel comfortable to disclose themselves, including their negative experiences in teaching, to us during interviews. For example, one participant told us that she recalled discarding students’ assignment when she did not have enough time to mark them. If the students asked her to return the assignments, she denied she had given them the assignments in the first place.

### Research Ethics

The researchers explained the research purpose, procedure, potential risks and benefits, and the participant’s rights in the study to all the participants before the study and obtained the participants’ written consents to ensure that their participations were voluntary. Moreover, no participants were coerced into participating in this study and all participants were allowed to withdraw from this study at any time. The participants also had the right to stop tape-recording during the interviews. In one interview, the participant requested a researcher to stop tape-recording of one of her teaching recollections because the story contained private information of a student. The tape-recording was stopped and replaced by note-taking until the participant expressed consent later for resuming the tape-recording.

Confidentiality is an important principle within an ethical research. To keep the records confidential, the researchers ensured that all the information collected was restricted only to the research team’s access. The researchers were careful not to disclose information, such as participants’ names and their school names, in the transcripts and final reports that may indicate the participants’ identity. Only pseudo names were used in reports in order to protect participants’ privacy.

The participants had the right to review the transcripts and the analysis through member check. Mero-Jaffe (2011) described member check as a necessary step to an ethical qualitative research because the participants can tell the researcher in the checking process where their identities may be released in the transcripts and the report, which parts of the transcriptions and interpretations may be unfair to them, and what information they may feel anxious about in case it were to be released to the public.

Finally, in order to make sure that the study conforms to the standards of research ethics, the study was reviewed and approved by a research ethics committee of a university in Hong Kong before its implementation.

## Results

### Caam^2^

Generally, the participants frequently expressed negative emotional experience and loads of complaints about their working condition during interviews. It seems that the participants did not enjoy teaching. This could be gathered from how they had reacted when talking about their working condition. For example, some of them kept sighing, some had rather trembling voices, and some even had tearful eyes. In terms of appearance, they looked weary and exhausted. For instance, some of them were sleepy and even fell asleep during the interviews. Many participants described their working condition as *caam^2^. Caam^2^* is a Cantonese adjective covers a range of meanings like gloomy, dreadful, tragic, pitiful, pathetic, and miserable. In other words, the participants’ expression reflected their emotional experiences in teaching tended to be negative in general.

### Workload

The data revealed that workload was the common reason why the participants felt *caam^2^* in teaching. All the participants complained that their workload was really heavy. For example, they said they always stayed at their schools from 8 a.m. to 6 p.m., although their official working hours generally were between 8 a.m. and 4:30 p.m. Going home did not mean an end to their work. They always continued to perform work tasks like marking and lesson preparation at home. When they finished the work, it might already be midnight. Sometimes, they might be unable to complete their work in the weekdays, so they had to sacrifice their weekends and holidays to continue with their work. This situation has led to the stress and exhaustion among many of the schoolteachers. The comment from Jack quoted at the beginning of the article is one example.

### Interpretations of Teachers’ Work

Moving from the emotional experience, we describe the participants’ interpretations of their work as teachers in this section, and will analyze the relationship between teachers’ emotional experience and interpretations in the next section.

#### Making a Difference

In general, the participants interpreted that teachers’ work was the endeavor to make a difference, i.e., nurturing students’ whole-personal growth. It was reflected in their reasons to becoming teachers.

“I love teaching because I think teaching is a meaningful job. You can teach someone to become a nice person spiritually and mentally. I mean I can help children to pursue their goals and dreams. Sometimes, I can even foster positive changes on naughty students.” (Crystal)

Indeed, some participants (e.g., David, Jack, Tom, and Isabella) did not have the interpretation before or at the beginning of their teaching career, because they regarded teaching only as a stable and secure job which offered them a high social reputation and salary. However, after they had interacted with students, they learnt that teaching meant helping students to learn and grow. Consequently, they discovered that teaching was a meaningful occupation and then changed their original interpretation of teachers’ work to that of making a difference and became committed to teaching.

Although the participants interpreted teaching as the endeavor to make a difference, they perceived that a lot of the actual teachers’ work was unrelated to education or did not facilitate them to make a difference. They categorized such work as “non-instructional work.” On the other hand, they referred the work which was related to education or could facilitate them to make a difference to “instructional work.”

#### Instructional Work

The participants agreed that the actual teachers’ work with respect to their teaching responsibility and the teacher–student interaction were instructional work in general.

First, teaching responsibility concerned not only with classroom teaching, but also lesson preparation and marking. It was found that the participants needed to teach five lessons on average per day (their schools had eight lessons every day in general) and to teach four different classes (a class had around 30–40 students) of four different grades. Many of them taught two to three subjects. Thus, they needed to prepare different materials and subject contents for different students at different grades, and mark many assignments after lessons. In fact, many participants, regardless of the subjects they taught and the years of teaching experience they had, said that lesson preparation and marking were time-consuming. Moreover, all the participants said that they were required to attend meetings, called collective lesson preparation, once a week. This meeting was organized in their free lessons or after school. In the meeting, teachers teaching the same subject and grade sat together and shared their teaching progress and experiences. In other words, the more subjects and grades teachers taught, the more such meetings they were required to attend.

Second, teacher-student interaction was another kind of instructional work to the participants. Teacher–student interaction was an important responsibility of teachers, because the participants believed that the interaction could help them better understand their students’ needs and enable them to facilitate students’ learning and growth. In order to have good interaction with students, they would take initiatives to approach students during recess, lunch hour, and after school. Many participants said that when they noticed students showing signs of behavioral or emotional problems, they would talk to them and counsel them in their free time in order to help them overcome their problems. Some of the participants even created a special Facebook account for their students. They would talk to the students, answer students’ questions, and encourage students to study through Facebook after school.

#### Non-instructional Work

First on the list, many participants mentioned that organizing extra-curricular activities (ECAs) was non-instructional. All the participants said that they were each responsible for at least one ECA in their schools. As a person-in-charge, each needed to take care of a lot of ECA-related administrative work, such as booking the venue for the activities, contacting service providers if necessary, and preparing notices about the activities for parents. If the ECAs were arranged outside regular school days, they might also take the students to the activities during their holidays.

In addition, the participants mentioned that they were asked to join one to two school committees/teams (e.g., guidance, career guidance, discipline, moral and civic education, and school administration and management) on average in addition to their subject departments. As committee/team members, they were responsible for the work assigned by the committees/teams. Since the nature and function of each team were different, it is hard to list all the work of each team. Nevertheless, some of the common work could be identified based on the interview data. First, all the committees/teams had many meetings in which the members discussed committee/team affairs. Second, all the committees/teams required their members to compile a lot of paper work and reports. Third, in many committees/teams, members were asked to organize activities or events for students, parents, and the schools. For example, teachers in a guidance committee and a moral and civic education committee would organize activities and programs to aid students’ personal growth and development.

Moreover, the participants suggested that some of their non-instructional work originated from performing special roles in addition to the teacher role in schools, such as homeroom teacher, grade coordinator, and subject panel head (director of a subject department) or committee/team leader. According to them, each of these roles involved many administrative duties. For example, homeroom teachers had to deal with many class affairs, contacts with parents, and student disciplinary matters; grade coordinators needed to hold collective lesson preparation meetings, evaluate the teaching and learning difficulties in that grade, and provide teaching support for other teachers; subject panel heads had to write the teaching progress plans for each grade, organize subject-related activities for students, be responsible for teacher appraisal, and prepare reports for the subject department; and team leaders had to write proposals or year plans for the team, set the team’s aims and policies, design programs to meet the aims, evaluate all the programs organized, and prepare reports.

### Emotional Experience of *Caam^2^* and Teaching Experience

Based on the analysis above, it appeared that the participants felt *caam^2^* in teaching because they needed to handle different kinds of instructional and non-instructional work. However, workload might just be the apparent reason of their feeling of *caam^2^* in teaching. A closer examination of the data discovered that the underlying reason was related to the interpretations of teachers’ work, especially on the interpretation of the instructional and non-instructional work. It became more obvious when comparing the narratives between teachers with different teaching experiences. To illustrate this point, the participants were categorized into three groups in terms of their teaching experience: early-career (≤6 years of teaching experience), mid-career (9–20 years of teaching experience), and late-career (25–40 years of teaching experience).

#### Early-Career Teachers

During the interviews, the nine early-career teachers expressed many complaints and exhibited negative feelings toward the working conditions described above. One reason was that they interpreted most of their work as purposeless to teaching.

As mentioned, these participants wanted to make a difference. Thus, they valued instructional work more than non-instructional work. For them, instructional work referred strictly to teaching responsibilities and teacher–student interaction. However, in reality, they believed that they had to do much non-instructional work that occupied most of their time. As a result, the early-career teachers felt *caam^2^*, because they perceived that they have spent a lot of time on non-instructional work.

“The current working condition makes me feel kind of frustrated. This is because I expect that teachers’ work should comprise mainly of lesson preparation, classroom teaching, and students’ problems solving, but now I need to do far more. Sometimes I can’t even spare time to prepare lessons and check students’ assignments. I don’t feel like I am devoting myself to my students. I don’t know what I’m doing.” (Mandy)

Although the teachers perceived most of their work as purposeless, they were in no power to change or reject the work.

“Our duties are assigned by the school at the beginning of the school year. All administrative duties are already assigned to me regardless of my willingness (sneering). We just can’t say no. We have to get it done.” (Amy)

This suggested that there might be a presumption in the teachers’ mind that they did not have the right to decline work assigned by the school, whether they valued the work. Another possible reason why they had little power was that they were generally temporary teachers. As Crystal stated, “as novices, we’re afraid of expressing opinions because our jobs are contract-based. We’re afraid of being fired. We’re afraid of saying something offensive to others resulting in the loss of our job.”

Accordingly, the early-career teachers experienced the feeling of *caam^2^* because they interpreted a lot of their work as purposeless, but they were powerless to refuse or change the work they had been assigned.

#### Mid-Career Teachers

The seven participants within this category were tenure teachers, except Jack, and four of them were subject panel heads. Like the early-career participants, the mid-career participants were committed to making a difference and preferred instructional work to non-instructional work. They also were dissatisfied with the non-instructional work that occupied most of their time.

“Sometimes I feel helpless, because I was forced to make the non-instructional work a top priority. I feel uncomfortable about this. As teachers, we really want to transfer our academic knowledge or life experience to the students. But does our work really allow us to do so? I feel particularly uncomfortable because I have no idea whether the students can learn through much of the administrative stuff on which we have spent tons of effort.” (Eva)

This comment suggested that even though the mid-career teachers were tenure and subject panel heads, they might still feel powerless to refuse non-instructional work. This might be because they were excluded from the school decision-making process.

“I feel quite helpless. I can neither change nor control the reality. It’s a great mission for every teacher to teach well. But the biggest problem is I’m not the one to make decisions. We are passive.” (Tom)

A small difference was noted between the mid-career and early-career teachers in the nature of their powerlessness. As we have seen, the early-career teachers were powerless because they perceived that they did not have the right to say no. Although the mid-career participants might also have the same interpretation, many of them, even if they were temporary teachers (e.g., Jack), attempted to express their opinions directly to the school administrators when they were dissatisfied with their working conditions. However, the administrators did not necessarily accept their opinions, and naturally their powerlessness was related to the administrators’ unsympathetic responses.

Nevertheless, the mid-career teachers might be able to cope better with the emotional experience of *caam^2^* than the early-career teachers. From the data, it could be seen that some of the mid-career teachers were able to manage their emotions by redefining non-instructional work. If they successfully redefined the work, they could have felt better and experienced less antagonism doing the non-instructional work. For example:

“At the beginning, I really disliked doing non-instructional work because I questioned what I was doing. However, later I found that I should learn to understand the meaning behind the non-instructional work and discover the possible positive outcomes brought to the students. Even though these are not really related to students’ academic performance, we need to figure out whether there are any components that may be beneficial to the students. If we find any, we will feel more comfortable.” (Eva)

#### Late-Career Teachers

Five late-career teachers in this study were tenure teachers. Three of them were committee/team leaders, while one was a subject panel head. Similarly, they faced the problem of heavy workload. They also disliked such working conditions. However, they expressed less negative feelings and complaints than the early- and mid-career participants in the interviews. A possible reason was that they had a different interpretation of their work. From the late-career participants’ points of view, instructional work and non-instructional work were closely related. For example:

“In fact, administrative work is kind of instructional work. I truly believe that all administrative work is ultimately aimed at educating students. Are guidance activities and school promotions instructional? Of course! That’s why I am the right person to handle these tasks.” (Sam)

In addition, the late-career teachers commented that teaching was a “rewarding occupation.” This might be because they had witnessed many students grown up throughout their teaching career and thus they perceived that they really did made a difference in students’ lives.

“Teaching is a mighty job. We can witness how a child grows. It’s great to see some of them eventually find their way and some of them gradually become very well-behaved. And after they graduated, all of them have entered into professions that suit them.” (Connie)

## Discussion

The findings suggest that although teachers may feel *caam^2^* in teaching because of heavy workload, the heavy workload might not be the underlying reason why the emotional experience of *caam^2^* arises. According to the appraisal theory, the underlying reason should be related to the instructional goals of teachers (Frenzel, 2014). If the teachers perceive that they can attain the instructional goals in teaching or that their work is conductive to the attainment of the instructional goals, they may feel positive; otherwise, they may feel negative (Frenzel et al., 2009; Frenzel, 2014; Beck et al., 2015). However, the teachers in this study did not mention any instructional goals when they talked about their emotional experiences of *caam^2^* in teaching. One possible reason is that the instructional goals were not the most significant goal in teaching among the teachers. As other studies illustrate, the most significant goal in teaching among teachers should be one that links with values and meanings of teaching (Lortie, 1975; Hargreaves, 1999; Isenbarger and Zembylas, 2006; Hansen, 2008). Thus, this study does not have enough evidence to illustrate how the social psychological process of teachers’ emotional experiences has involved instructional goals.

On the other hand, the study may provide supports to social constructionism of emotions. According to the perspective, emotions are aroused by the interpretation of the self or the situation (Denzin, 1984; Turner, 2007). Similarly, the findings imply that the emotional experience of *caam^2^* may be aroused by the teachers’ interpretations of their work. The findings further suggest that there are two levels of the interpretations of teachers’ work. The first level is the general meaning of teaching. As other researchers (Lortie, 1975; Santoro, 2011; Saunders, 2013) have noted, the study identifies that teachers may associate the meaning of making a difference to teaching. Since they commit themselves to the meaning, as predicted by social constructionism, making a difference may become a significant goal in which they would like to attain in teaching. Moreover, the goal also implies that the teachers may define themselves as moral change agents who would devote themselves to “changing” the students (Hansen, 1998, 2008). Therefore, when they think they fail to make a difference, they may interpret themselves as impoverished, incompetent, and even immoral teachers resulting in the emotional experience of *caam^2^*. The findings are similar to the studies conducted by Kelchtermans (1996, 2011) and Farouk (2012), which find that teachers may experience intense negative emotions, such as depression, frustration and guilt, because of the failure or difficulty to facilitate students’ whole personal growth. It is noted that the teachers’ interpretation of whether they attain the goal of making a difference may be related to the second level interpretation of teachers’ work, which is the meaning teachers give to the actual teachers’ work. At this level, teachers categorize their actual work as either instructional or non-instructional work. It is found that the emotional experience of *caam^2^* may occur if the two levels of meanings are inconsistent. That means teachers may feel *caam^2^* in teaching, if the teachers perceive most of their work as non-instructional work which is contradictory to the significant goal of making a difference.

What is more complicated is that the interpretations of teachers’ work, especially those at the second level, may be different among teachers who have different teaching experiences. As the findings illustrate, the less experienced teachers tend to view most of their work as non-instructional, but the experienced teachers tend to view all of their work as instructional, or at least partially instructional. Why is there a difference? According to social constructionism, it may be related to power (Turner, 2007, 2011). The research findings suggest that power is unequally distributed among teachers according to teaching experiences and their managerial roles, and teaching experiences and managerial roles may be the interlocked factors. In this study, most of the late-career teachers were appointed as committee/team leaders in schools. In Hong Kong secondary schools, the committee/team leaders are the school administrators who assist the principal to manage the school (Cheung and Kan, 2009). Thus, these teachers had stronger power that enabled them to better understand the values of teachers’ work assigned by the school. On the other hand, the less experienced teachers, even though they were subject panel heads, generally had limited power in determining classroom activities or subject-related issues (Wong, 1997). Therefore, it was hard for them to reject the overwhelming duties assigned by the school, especially those administrative duties of which the underlying values, if any, could not be well comprehended by the less experienced teachers. As a result, they may think that they are assigned to spend time on many purposeless tasks or roles but are powerless to change the situation. This condition may lead to the emotional experience of *caam^2^*. In other words, the interpretations of teachers’ work as well as the emotional experience of *caam^2^* may be structurally distributed among teachers at an unequal extent according to the teachers’ teaching experiences, managerial roles, and power.

The above discussion suggests that the social psychological process of teachers’ emotional experience may involve the following components. First, teachers may define and agree teaching as an endeavor to make a difference which becomes their significant goal in teaching. Thus, they may aspire to pay attention to instructional work. If they interpret most of their actual work in teaching are non-instructional, they will feel negative. This is because they may perceive that the working condition cannot effectively help them to attain the goal of making a difference. Second, teaching experience may affect the interpretations of teachers’ work. More experienced teachers tend to interpret much of their actual work as instructional, but less experienced teachers tend to interpret much of their actual work as non-instructional. Thus, more experienced teachers may feel less negative than less experienced teachers. Third, teachers with power will feel more positive than teachers without power or with limited power in school. The reason is that the teachers with power is more able to identify the instructional value of the work, especially administrative duties, than the teachers without power or with limited power, since the teachers with power are involved in the school decision-making process while the teachers without power or with limited power are not. Fourth, power is positively related to teaching experience, i.e., more experiences in teaching, stronger the power.

The research findings have some implications. First, teacher education programs should encourage teachers to explore and understand the importance and value of their work, including those working tasks that they perceived as non-instructional. Many teacher education programs focus on technical issues in teaching, such as subject knowledge, teaching approaches, classroom management, and curriculum development (Hansen, 2008). However, the concept of teaching should go beyond these technical issues. For example, it may be unhealthy for students to only learn subject knowledge inside classrooms without joining any ECAs. Similarly, a successful education is not only about classroom teaching, but is also dependent on many other administrative duties behind teaching. Thus, teacher education programs should go beyond training technical skills of teaching. It should invite the teachers to appreciate the importance of the so-called non-instructional work in teaching.

Second, school administrators should empower teachers, especially those with less teaching experience or less managerial roles. This is because the teachers without power or with limited power may experience difficulty in understanding the instructional values of their work which may result in their negative emotional experience. To empower teachers, it is suggested that schools should allow more teachers to participate in the school decision-making process or consult teachers’ opinions more openly before making school decisions. The practice may not only give the teachers a sense of ownership of their work, but also enhance their understandings of their work (Leithwood and Beatty, 2008).

Indeed, some teachers may be asked to do a lot of administrative duties with limited instructional value, draining away their time and energy, and obstructing them from paying attention to students’ learning and growth (Hargreaves, 2003). Therefore, it is suggested that school administrators and education policy makers should consider freeing the teachers from their heavy administrative workload. For example, they may increase the administrative manpower to handle the administrative duties. Not only it may improve teachers’ emotional experiences, but it may also enhance the quality of the education.

One limitation of the study is sampling bias. As mentioned in Section “Researcher Reflexivity,” most of the participants were the authors’ friends or friend-referred friends. Therefore, the findings may not fully represent the patterns of teachers’ emotional experiences in general. Moreover, the study only qualitatively investigated 21 secondary schoolteachers in Hong Kong. Therefore, the findings may not be generalizable to a large population of teachers, especially the primary schoolteachers and the teachers in other cities. In order to overcome the limitation, it is suggested that further research may attempt to validate the findings of the present study with different groups of teachers by applying probability sampling strategies. Hypotheses may also be generated based on the research finding, for example: (a) the more teaching experiences, the more positive teachers’ emotional experiences; (b) if teachers have more important administrative roles, they will feel more positive in teaching; (c) if teachers interpret most of their work as non-instructional, they will feel more negative in teaching. Then, the hypotheses can be tested by survey method with a larger sample size.

## Author Contributions

KT makes substantial contributions to the conception and design of the work and the acquisition, analysis, and interpretation of data for the work. TK makes contributions to revision of the work critically for important intellectual content.

## Conflict of Interest Statement

The authors declare that the research was conducted in the absence of any commercial or financial relationships that could be construed as a potential conflict of interest.

## Footnotes

1 All names have been changed to preserve anonymity.
